# Why Being ‘Stressed’ Is ‘Desserts’ in Reverse—The Effect of Acute Psychosocial Stress on Food Pleasure and Food Choice

**DOI:** 10.3390/foods11121756

**Published:** 2022-06-15

**Authors:** Nikoline Bach Hyldelund, Vita Ligaya Dalgaard, Derek Victor Byrne, Barbara Vad Andersen

**Affiliations:** 1 Food Quality Perception and Society Team, iSense Lab, Department of Food Science, Faculty of Technical Sciences, Aarhus University, 8000 Aarhus, Denmark; derekv.byrne@food.au.dk (D.V.B.); barbarav.andersen@food.au.dk (B.V.A.); 2 Sino-Danish College (SDC), University of Chinese Academy of Sciences, Beijing 101408, China; 3 Department of Psychology and Behavioral Sciences, Aarhus University, 8000 Aarhus, Denmark; ligayadalgaard@psy.au.dk

**Keywords:** food pleasure, food reward, stress, Leeds Food Preference Questionnaire, wanting, liking

## Abstract

The link between acute stress, food pleasure and eating behavior in humans by employing measures of individual reward mechanisms has not been investigated as of yet. Having these insights is key to understanding why many people experience a change in eating behavior when experiencing stress. Thirty-five Danes (mean age 21.71 years) underwent a stress-inducing and relaxation-inducing task based on a randomized cross-over study design. Both tasks were combined with the Leeds Food Preference Questionnaire, to investigate the effect of stress on specific measures of food reward. Furthermore, participants chose a snack, as a covert measure of actual food choice. The study found no effect on explicit liking, explicit wanting or relative preference. For implicit wanting, an effect was detected on high-fat sweet foods, with increasing scores for the stress-induced condition. Moreover, 54% chose a different snack following the stress-inducing condition. Interestingly, 14% chose to change their snack choice to no snack at all. Results suggest acute psychosocial stress can increase cravings for highly palatable foods for some, while for others an experience of loss of appetite prevails. Overall, this study points to a further understanding of why consumers have issues with making healthy food choices, ultimately affecting public health too.

## 1. Introduction

The German sociologist Hartmut Rosa has described the paradox of how modern work life has been organized [1]. On one hand, modern work life seeks to accommodate higher levels of individual freedom, and on the other hand, it has been associated with increased levels of perceived stress, and health issues are registered as a result of working under these conditions [1,2,3,4]. Rosa describes this paradox as a result of a phenomenon that he has termed ‘social acceleration’ [1,2]. Social acceleration is a theoretical expression of how the pace of all parts of life constantly speeds up, leaving people with a feeling of not being able to keep the pace, feeling insufficient, and as a result, some may experience increased stress or burnout. Prolonged exposure to stressful conditions has been linked to a long and varied list of clinically defined diseases. These links are mediated by both the neuroendocrine systems, alterations of health behavior and affective regulation, which potentially can result in diseases such as depression, cardiovascular diseases, diabetes, infectious diseases and neurodegenerative diseases [5,6,7,8,9].

### 1.1. Physiological Mechanisms of the Endocrine System Affected by Stress

The most central physiological stress response is the activation of the hypothalamic-pituitary-adrenal (HPA) axis, which begins with the release of corticotrophin-releasing factor (CRF) from the hypothalamus [10,11,12,13,14,15,16,17]. CRF stimulates the release of adrenocorticotropic hormone (ACTH) from the anterior pituitary gland. ACTH then circulates through the bloodstream to the adrenal cortex, where it stimulates secretion of glucocorticoids, such as cortisol. Glucocorticoids (GC) can promote eating and weight gain in two ways. First, GC in rodent models has been shown to stimulate eating, especially highly palatable foods, on its own [14,18]. Secondly, GC stimulates insulin secretion, and the two hormones can then further act synergistically to promote food intake and visceral fat accumulation, as GCs functions to increase general food-associated drives, while insulin influences preferences for which specific types of food are consumed [14,16,19]. Additionally, cortisol will bind to GC receptors and thereby activate lipoprotein lipase, which may increase triglyceride accumulation in fat tissue, especially in the abdominal region. In the presence of insulin, cortisol will also concurrently inhibit the lipid-mobilizing system, which can also result in further fat accumulation. The HPA axis is thus not only the manager of the stress response but is also highly intertwined with the endocrine regulation of appetite, as the hypothalamus is a critical region for regulation of both food intake and energy balance and the stress circuit [10,11,14,16,20]. Furthermore, stress also activates the autonomic nervous system (ANS), which may result in heightened sympathetic-nervous-system activity and subsequent release of adrenaline and norepinephrine. It is especially these hormones that activate the allostatic stress response, and in synergy with the glucocorticoids starts a series of adaptive processes that can alter the structure and function of a variety of cells and tissues [8,21]. Furthermore, ongoing high levels of experienced stress can also result in overactivity of the sympathetic nervous system, which is related to insulin resistance [16]. The ANS may therefore in part be responsible for the higher prevalence of cardiovascular diseases, metabolic diseases and changes of the immune system that are seen among patients suffering from stress [8,9,21,22].

### 1.2. Stress-Induced Eating

There are two different, yet interacting, pathways of the physiological response to acute stress that can affect food intake. The first is the activation of the HPA axis, with subsequent stimulation of the secretion of glucocorticoids (including cortisol) as described above. The other pathway is that of the sympathetic nervous system, which leads to increases in arousal parameters such as secretion of adrenalin, elevated blood pressure and a diversion of blood flow from the gastrointestinal tract towards the skeletal muscles and brain [16,20]. This reaction is also known as the ‘fight-or-flight’ response and will most often lead to a decrease rather than an increase in food consumption. Nevertheless, research has shown that if the stressor is perceived as ego-threatening, i.e., as a threat to self-esteem or understanding of social self, cortisol will be released, and thereby stimulation of appetite and food intake will follow [20,23].

Multiple studies have shown that being stressed, both by acute stressful events as well as during longer periods due to interpersonal and work-related daily hassles, can cause non-homeostatic hunger, as food intake can dampen the physiological and behavioral stress responses [12,14,19,24]. The physiological stress response can be dampened by eating, as the secretion of ACTH is reduced following consumption of food and the activation of the HPA axis is thereby attenuated [16,18,19]. The relation between stress and food intake can therefore be characterized as bidirectional, as stress and negative effects can alter eating behavior by increasing intake of especially highly palatable foods via activation of the HPA axis. Conversely, food consumption can alter mood by attenuating the stress response via the endocrine system and hedonic effects of the food. In particular, the hedonic experience of eating has been proposed to serve a special role in the effect of stress-induced eating, as eating can activate neural substrates, such as dopamine, similarly to drug abuse [5,16,20,25,26]. Dopamine is a neurotransmitter that codes for pleasure and enhances the desire for food, while it also functions to deactivate the HPA axis activity [16,27,28]. The pleasure one experiences from food may therefore be one of the main reasons for the comforting effect of food when feeling stressed. Thereby, by eating as a means to dampen the physiological and behavioral stress response, the reward pathways are concurrently stimulated, which can potentially lead to neurobiological adaptations that promote the compulsive nature of overeating in a way that resembles drug abuse [20]. GCs are furthermore involved in the regulation of memory, specifically, those memories that are consolidated of emotionally arousing experiences, such as stress [18,19,29]. Thereby, as stress promotes secretion of GCs, which may lead to intake of highly palatable foods, the GCs at the same time can facilitate an association between the indulgence of these ‘comforting’ foods and a subsequent positive effect on stress and mood [14,16,20,30].

### 1.3. Food Reward and Its Subcomponents

In addiction research, there is a sharp distinction between ‘liking’ and ‘wanting’ as two key components in food reward [25,26,31]. Liking, or consummatory pleasure, is linked to the hedonic reaction to a food, and can be detected by behavioral or neural signals in hedonic hotspots in the brain. Wanting, or incentive salience, on the other hand, relates to the motivation for a reward, typically triggered by a reward-related cue [25,26,32]. ‘Wanting’ is generated by large dopamine-related mesolimbic brain systems, and will ordinarily occur together with ‘liking’ and learning. However, as the ‘liking’ and ‘wanting’ systems are separate from each other, both mechanisms can also occur independently from the other [25,26,33,34,35]. A well-known mechanism from drug addiction is the case of experiencing compulsive levels of ‘wanting’ without ‘liking’, as a consequence of sensitization of the reward pathway [19,25,35]. It is hypothesized that this mechanism is what may be predominant in the case of food addiction too [26,27,33,34,36]. The link between the stress response, stress-related eating and behavioral changes thus seems to be closely related to the reward pathway, as well as changes in the mesolimbic system due to increased dopamine secretion or dopamine sensitization [16,26,35,37,38].

### 1.4. Purpose of the Current Study

A vast part of stress-eating research is based on animal studies, as only these studies can provide the essential causal data of the brain-based mechanisms without violating ethical regulations [10,19,26,32]. Of such studies, rodent models have demonstrated how comfort eating can reduce the activation of the HPA axis [10,11]. Nonetheless, human studies can provide insights into the emotional processes of stressful eating, and can as such offer psychological and behavioral perspectives to further understand the brain mechanisms involved in both the stress responses and eating behavior as a result thereof [14,18,20]. Previous research has found an unambiguous relationship between chronic stress, altered eating behaviors and obesity [16,24,39]. However, it is not clear yet whether changes in perception of food reward is exclusively manifested in chronic stress conditions, or whether this affect can also be observed in an acute stress condition. To the authors’ knowledge, no studies have thus far investigated the link between acute stress and food reward in a human study by combining both physiological, subjective and behavioral measurements. By doing so, the ‘stress-eating behavior’ relationship can be explored, not only by consumption levels or food choice of specific food groups, but by the underlying components of anticipatory and experienced food pleasure; explicit liking, explicit wanting and implicit wanting. Having these insights could be the key to further understanding why many people in postmodern society experience continued loss of control of their diet. The overall aim of this study is to investigate the link between acute psychosocial stressors, perceived pleasure from food and consequently food behavior in humans to fill the scientific gap described above. Specifically, the objectives of the study are to:

- Investigate how explicit food liking, explicit food wanting, implicit food wanting, and relative food preference are affected by temporary acute stress vs. relaxation.
- Explore if actual snack food choice is affected by temporary acute stress vs. relaxation.

Regarding the first objective, it was hypothesized that explicit liking and relative food preference would not be affected by the stressful condition, as these reward measures reflect a more stable side of hedonic perception. Oppositely, it was hypothesized that explicit wanting and implicit wanting would increase for the calorie-dense food categories, as wanting is regarded as a more context-dependent measure, and in the context of acute stress, increased wanting can be a symptom of pronounced sensitization of the reward pathway. In addition, previous research has found correlations between stress and increased consumption of highly palatable foods [16,20]. Therefore, actual snack choice was expected to reflect explicit liking and wanting, and thus also turn towards the more calorie-dense snacks after completion of the temporary acute stress-inducing task.

## 2. Materials and Methods

### 2.1. Study Design

Participants underwent two different tasks based on a cross-over study design—one task that induced psychosocial stress and one task designed to keep the participant in a calm neutral state. Thereby, each participant served as their own control. Both tasks were combined with a subsequent food preference test, the Leeds Food Preference Questionnaire [25,33,40], to investigate the effect of stress on food choice, explicit liking, explicit wanting and implicit wanting as measures of both anticipatory and experienced food pleasure. The order in which the participants performed the tasks was randomized. For ensuring reliability, avoid carry-over effects and to control for external factors, such as tiredness, the two tasks were performed on separate days.

### 2.2. Participants, Recruitment and Prescreening

Thirty-five healthy Danes were recruited via the Danish research recruitment site (www.forsøgsperson.dk [Edit., ‘Eng: www.testsubject.dk’], accessed on 1 November 2021), as well as online social media posts on Facebook. A prescreening process was performed to ensure the participants were of a somewhat homogeneous group, and that they each met a set of pre-defined inclusion and exclusion criteria. The inclusion criteria were; 18–50 years of age, fluent Danish language as well as a BMI within the ‘normal-weight’ range (BMI: 18.5–24.9). The exclusion criteria were; Current restrictive dieting or adherence to a very specific diet, history of substance abuse or having eating disorders, current diagnosed psychiatric condition, current and/or a history of treatment for chronic stress, as well as metabolic or endocrine diseases. The exclusion criteria were chosen based on incompatibility with the study methods and ethical considerations. A questionnaire was developed for use in the screening process to ensure all criteria were met. Characteristics of the participants can be seen in Table 1. Ethical approval of the study protocol was obtained from the Health Research Ethics Committee of Central Denmark Region prior to recruitment (Case number 1-10-72-294-21), and the study was conducted in accordance with the ethical standards laid out in the Declaration of Helsinki [41]. All respondents gave written consent to use of their data prior to commencing the questionnaire. On the test days, the participants were instructed to have a good night’s sleep, defined as 6–8 h of continuous sleep, as well as to go to sleep at similar times the night before each test day. Furthermore, they were asked to (1) not smoke 3 h prior, (2) not engage in any moderate-to-vigorous exercise 2 h prior, and to (3) consume a meal that leaves them comfortably satiated. The researcher verbally confirmed with the participants that they adhered to these instructions. At the onset of the first test day, the participants were asked to provide written consent. All participants were fully debriefed and paid for their participation after the second test.

#### Power Calculations

A power analysis was completed based on results of previous research using the Leeds Food Preference Questionnaire [33]. The calculation was grounded on the mean frequency of food choices for the food category ‘high-fat sweet’, before and after a meal. A power calculation by use of the two-sided *t*-test at the 5% level for continuous data was performed to estimate sample size. It was found that a sample of 28 participants with a power of 80% could find a difference of 15 in a distribution with a standard deviation of 14.51. A sample of 35 individuals was therefore pursued to ensure statistical power.

### 2.3. Study Procedure

**Figure 1:**
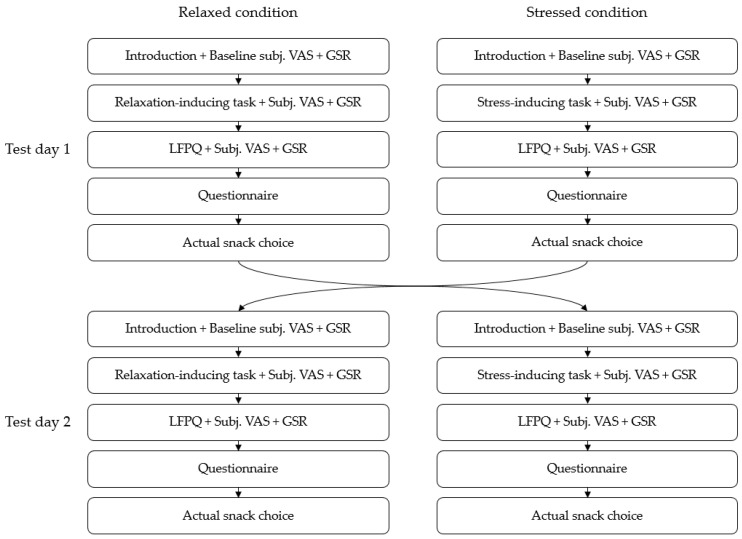
Flowchart of experimental procedure. The study followed a randomized controlled trial design, where each participant completed the Leeds Food Preference Questionnaire twice—once after completion of the relaxation-inducing task, and once after completion of the stress-inducing task. Subjective measures by visual analogue scales, GSR, a self-report questionnaire and actual snack choice was likewise recorded for both test days.

Figure 1 illustrates the complete study design and test variables. After providing written informed consent at the onset of the first test day, the participant was introduced to the procedure of the study as well as given instructions on how to perform the different tasks. Furthermore, Galvanic Skin Response (GSR) leads were placed on the fingers of the participant’s nondominant hand, and the participant was instructed to limit movement of that hand as much as possible. GSR measurements were recorded as a secondary exploratory measure for the manipulation check. The participant was then asked to evaluate baseline levels of stress, affect, satiation and rest by a 100 mm visual analogue scale. Next, the participant was asked to perform a cognitive task, designed to induce either stress or relaxation. Immediately after performing the task, they were asked to assess subjective stress, emotional, rest and satiety levels once again, followed by the Leeds Food Preference Questionnaire and a final assessment of current subjective sensations. Subjective measures, instructions for cognitive tasks and the LFPQ was all programmed on the Gorilla Experiment Builder platform (www.gorilla.sc, 2022, accessed on 1 March 2022) [44], thereby allowing for online collection of behavioral data, including reaction times. Finally, a questionnaire was administered to collect demographic information and assessment of general stress level. The questionnaire was administered via the CompuSense^®^ Cloud software, Version 22.0.112022/03/03 (CompuSense Inc., Guelph, ON, Canada) [45]. The second test day followed the exact same procedure, although the cognitive task consisted of the opposite task as of test day 1. Debriefing regarding the purpose of the study, as well as data use, was performed after completion of the second test day to make sure all participants understood the purpose of the two cognitive tasks.

### 2.4. Measure of Food Reward and Eating Behavior

The Leeds Food Preference Questionnaire (LFPQ) is a computerized behavioral task that provides measures of ‘liking’ and ‘wanting’ components of food preference and food reward. The LFPQ have been found to provide valid and reliable measures of several aspects of food reward including explicit liking, explicit wanting, relative preference and implicit wanting for food categories of common foods in the diet [25,33,40,46]. The food categories were characterized as ’high-fat sweet’ (HFSW), ’low-fat sweet’ (LFSW), ’high-fat savory’ (HFSA) and ’low-fat savory’ (LFSA). The LFPQ consisted of two subtasks that required interactions from the participant. One task involved an explicit evaluation of food images from an array of prevalidated photographs using visual analogue scales and was used to measure explicit liking and explicit wanting. The other subtask required a rapid choice to be made between paired combinations of the food images of the different food categories, based on most-wanted food item at that specific moment, and was used to measure relative preference and implicit wanting. The order of the tasks was randomized within the program [40,46,47].

#### 2.4.1. Food Images in the LFPQ

Foods were chosen from a validated database to be either high or low in fat, sweet or savory (nonsweet) taste, and similar in familiarity, protein content and palatability, as well as validated in terms of being appropriate for Danish food consumers [47,48,49]. An overview of the foods depicted in the images and their respective macronutrient content can be seen in Table 2, and specific images can be seen in Table S1.

#### 2.4.2. Single Foods—Explicit Liking and Wanting in the LFPQ

In the single foods task, the participants were shown a single food image and were then asked to explicitly rate their expected liking (“How pleasant would it be to taste this food now?”) or wanting (“How much do you want some of this food now?”) on a 100 mm VAS scale. In total, 32 ratings were completed per participant—16 for explicit liking and 16 for explicit wanting. The two questions had different font colors to help the participant better discriminate between them. Moreover, a ‘break’ screen appeared after half of the questions had been answered, to give the participants an optional break from the continued demand of the task.

#### 2.4.3. Paired Foods—Food Choice and Implicit Wanting in the LFPQ

In the paired foods task, the participants were presented with a series of food image pairs and the instruction “Which food do you most want to eat now?”. Furthermore, the participants were instructed to make their choice as fast as possible (by use of the ‘F’ key for left choice and the ‘J’ key for right choice). The task presented all 96 possible pairs in a random order, in such a way that all food images from one food category were presented with each food from the other categories, with a ‘break’ screen inserted after every 32 trials to avoid response fatigue. Before each trial a fixation cross was shown for 500 ms to enable visual centralization. Mean frequency of choice of the four different food categories thus represents relative preference (food choice) for each of the food categories. Furthermore, reaction times for each trial were measured as a means for computing a measure of implicit wanting. In line with previous studies using the LFPQ implicit wanting score was calculated for each food category as a composite score for one food category relative to the other categories [40,47,50]. The score was calculated using the following algorithm and was based on frequency of choice, reaction time for both chosen and nonchosen foods, and a mean reaction time [40,47]. A positive score would indicate a higher preference for that food category compared to the others. Oppositely, a negative score would indicate a lower preference, whereas a score of zero would indicate equal preference.

$$Implicit\;wanting:I_{A}=\overset{N_{choice}}{\underset{i=1}{\sum }}\frac{\bar{t}}{t_{i}}-\overset{N_{non-choice}}{\underset{j=1}{\sum }}\frac{\bar{t}}{t_{j}}\;$$

Formula legend:

*I_A_* = Implicit wanting for category *A*;

*N_choice_* = number of times category *A* was chosen;

*N_non-choice_* = number of times category *A* was not chosen;

$\bar{t}$ = mean of all reaction times.

#### 2.4.4. Actual Snack Choice

**Figure 2:**
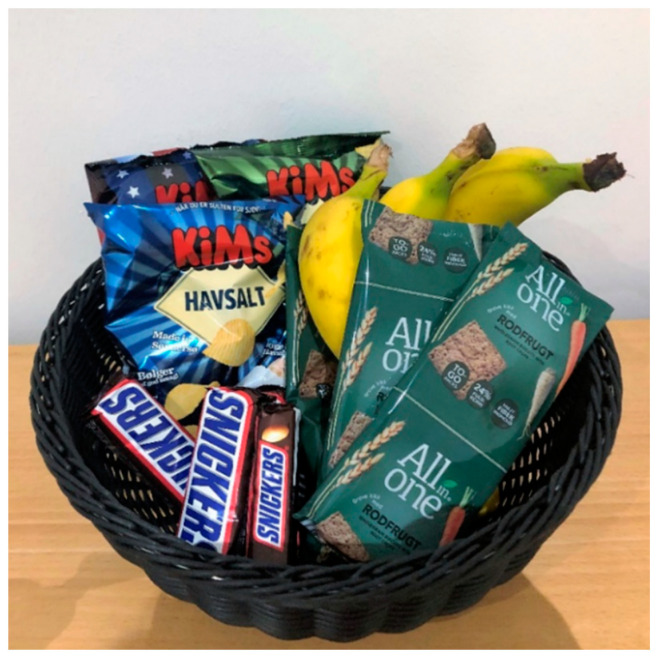
Selection of snack products as presented to study participants.

Actual snack choice was recorded by the researchers as a measure of implicit food choice. Participants were offered a snack after completion of each test day as a small ‘thank you’ for their effort. The selection of snacks represented the four food categories of the LFPQ, namely potato chips (Kims, Orkla Confectionery & Snacks Danmark A/S) = HFSA; chocolate bars (Snickers, Mars, Inc.) = HFSW; wholegrain crackers (All-in-one, Bisca A/S) = LFSA; and bananas (Cavendish, Fyffes Tropical Ltd.) = LFSW. Figure 2 shows the snack selection as presented to the participants, and Table 3 shows the macronutrient content of the snacks. Meticulous care was given to ensure that the snack selection was identical for each participant.

### 2.5. Cognitive Tasks

The stress-inducing task consisted of an ‘unsolvable anagram’ task, which has been designed to induce acute ego-threatening stress by making the participants believe they are failing a simple cognitive task [51,52,53]. The stress task procedure follows the design of Polivy and Herman, 1999. The participant was given a list of six 5–6-letter solvable anagrams, and six 7–8-letter anagrams, unsolvable except for the first two, to ensure that none will be able to complete the task. The participants were told that the task was a measure of cognitive function, with the purpose of investigating the effect of different cognitive abilities on personal food preferences. Furthermore, they were informed that the first 5–6-letter anagrams could be solved by most primary school students, and the latter 7–8-letter anagrams could be solved by most high school students. The participants were then instructed to solve the anagrams within 10 min, with a timer in front of them. After 10 min, the participants were instructed to proceed with the rest of the tasks (the LFPQ and the self-report questionnaire) (See Figure 2).

The relaxation-inducing task consisted of having the participants do a simple Mandala coloring page, to keep the participants in a relaxed, neutral state. Coloring has proven to be able to significantly reduce anxiety and increase mindfulness [54,55]. The participant had 10 min for coloring to mimic the time spent during the stress task, and after 10 min, the participants were instructed to proceed with the rest of the tasks (See Figure 2).

### 2.6. Subjective Sensations and Self-Report Questionnaires

At specific time points throughout the test days (see Figure 1), the participants were asked to indicate how stressed they felt at that specific time on a 100 mm-line visual analogue scale anchored by ‘None’ and ‘As bad as it could be’. The scale thus yielded a single subjective stress score between 0 and 100. This specific method of assessing stress have been validated and proven to be able to detect subjective levels of stress at an equally discriminating level as other more complex and time-consuming stress-assessment tools [56] such as the Perceived Stress Scale [42] and the Hospital Anxiety and Depression Scale [57]. Feeling hungry, emotional or tired is believed to cause symptoms similar to those of stress, and thus these factors were attempted to be controlled for by the participant instructions given prior to testing. To check for participants being affected by emotional states or not being fully rested or satiated on the test day, they were asked to assess, in a similar manner to the subjective stress, on a 100 mm-line VAS, to which degree they felt satiated, to which degree they felt emotional and to which degree they felt rested. All VAS scales were anchored by ‘Not at all’ and ‘Very much’ at the extreme ends.

A questionnaire to be filled out at the end of each test day was constructed. The purpose of the questionnaire was to collect information on demographic, anthropometric, as well as general stress level. The following self-reported demographic variables were included: age, gender, educational level and socioeconomic status. Participants were furthermore asked to state weight and height, and BMI was calculated for each participant using the standard formula of weight (kg)/height^2^ (m). A validated Danish version of the 10-item Perceived Stress Scale (PSS-10) was also incorporated in the questionnaire to assess the general stress level of each participant [43]. The PSS-10 is a global stress measure developed to assess the extent to which an individual finds their life to be unpredictable, uncontrollable and overloaded [42,43]. The PSS-10 consisted of ten questions regarding emotions and thoughts experienced within the last month. The participants were asked to assess how often they have experienced that specific emotion or thought in question, by a 5-point scale. Ratings of all items were averaged to create a mean score, with higher scores indicating a higher level of perceived stress.

### 2.7. Physiological Arousal

Tonic electrodermal activity (micro-Siemens, μS) was recorded as a secondary and exploratory measure of arousal levels during the test-day procedures. This was performed via the Biopac MP 150 Data Acquisition System (BIOPAC Systems Inc., Goleta, CA, USA) [58], the GSR100C unit and disposable BIOPAC EL507 electrodes with isotonic gel attached to the subjects’ nondominant hand fingers. The sampling frequency was 2000 Hz. A monitor and a standard computer mouse were peripherals for interaction between participants and a PC running the tasks, as well as performing the ‘Stress’ or ‘Relaxation’ task by the dominant hand of the participant with a pencil on a physical piece of paper. Data were analyzed with Acknowledge5^®^ software (BIOPAC Systems Inc., Goleta, CA, USA) [58]. Before analysis, the signals were filtered for noise reduction with a median smoothing factor of 20, as well as a 0.05 Hz high-pass filter was used to derive a phasic signal from the tonic signal. Skin-conductance responses were identified by use of the ‘Derive phasic EDA from tonic’ option in the software, with a threshold of 0.01 μS. Mean skin-conductance levels as well as peaks per minute were calculated specifically at a 1 min baseline period, during the 10 min cognitive tasks as well as during the performance of the LFPQ, to evaluate the changes in arousal throughout the testing.

### 2.8. Data Analyses

Data were exported from Gorilla Experiment Builder (www.gorilla.sc, 2022, accessed on 1 March 2022) [44] and CompuSense (CompuSense Inc., Guelph, ON, Canada) [45], as well as Acknowledge5^®^ (BIOPAC Systems Inc., Goleta, CA, USA) [58] for the GSR data. All data analyses were executed in R Studio©, version 1.3.1093 (RStudio Team, Boston, MA, USA) [59]. Significance level was set to α = 0.05. Calculations of descriptive statistics were conducted for all variables to obtain a complete overview of distributions and mean values. Differences between the two conditions in rating of subjective emotional state and stress levels as well as GSR measures were analyzed by linear mixed-effects ANOVA models, all adjusted for test day, level of restedness and satiation. Correlation tests between subjective stress levels and GSR measures were conducted as part of the manipulation check. To check if differences in rating by taste (sweet vs. savory) or fat content (low-fat vs. high-fat) appeared within the two different conditions, repeated measures ANOVAs were utilized. Effect of the two conditions (stressed vs. relaxed) on ‘explicit liking’, ‘explicit wanting’, ‘relative preference’ and ‘implicit wanting’ for each of the four food groups of the LFPQ were analyzed by Wilcoxon’s matched pairs signed rank tests, and effect sizes (r) were calculated if significant differences were detected. Finally, the effect of the two conditions on actual snack choice was analyzed by McNemar’s test. The effect of test-day order on actual snack choice was likewise analyzed by McNemar’s test.

## 3. Results

### 3.1. Manipulation Check on Stress and Emotional Level

**Figure 3:**
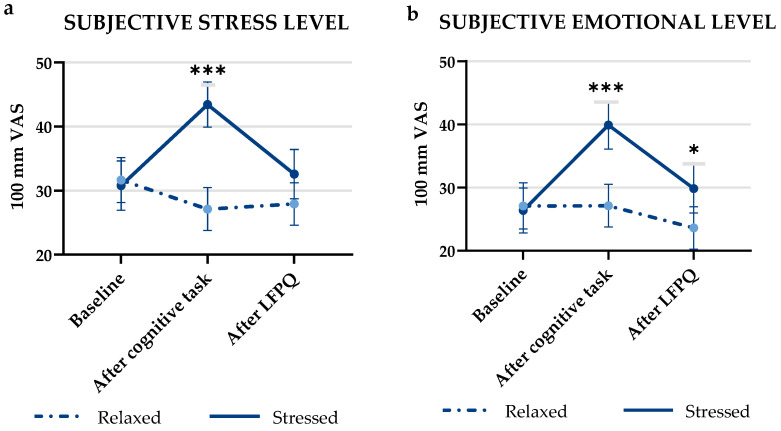
(**a**) Mean (±SEM) subjective stress levels measured at baseline, after the cognitive task and after performing the Leeds Food Preference Questionnaire (LFPQ), rated on a 100 mm visual analogue scale. (**b**) Mean (±SEM) subjective emotional levels measured at baseline, after the cognitive task and after performing the Leeds Food Preference Questionnaire (LFPQ), rated on a 100 mm visual analogue scale. Stars indicate level of significance of *p*-values. *: *p* < 0.05, ***: *p* < 0.001.

The stress-inducing cognitive task was perceived as being significantly more stressful by the subjective VAS scale (mean rating ± SD = 43.43 ± 20.88) than the relaxation-inducing task scale (mean rating ± SD = 27.11 ± 19.82; *p* < 0.001). After performing the LFPQ, no differences could be detected, and the stress level had dropped to baseline level for both tasks. Likewise, the participants reported to be significantly more emotionally affected after performing the stress-inducing task (mean rating ± SD = 39.89 ± 22.57) vs. performing the relaxation-inducing task (mean rating ± SD = 27.11 ± 20.03; *p* < 0.001). Similar results were found for emotional levels after performing the LFPQ, with higher levels of emotional arousal for the stressed condition (mean rating ± SD = 29.83 ± 22.71) vs. the relaxed condition (mean rating ± SD = 23.60 ± 19.89; *p* = 0.024). Figure 3a,b illustrate changes in rating of stress and emotional state during the test procedure for both conditions.

No significant differences in electrodermal activity levels were detected for the two conditions at either baseline, after the cognitive tasks nor after the LFPQ. Furthermore, no correlations between mean GSR levels and the subjective ratings of stress at baseline, after completion of the cognitive tasks and the LFPQ were found. Table S2 gives an overview of the results of the GSR measurements.

### 3.2. Eating Behaviour and Food Choice by Different Reward Measures

#### 3.2.1. Explicit Liking and Wanting

**Figure 4:**
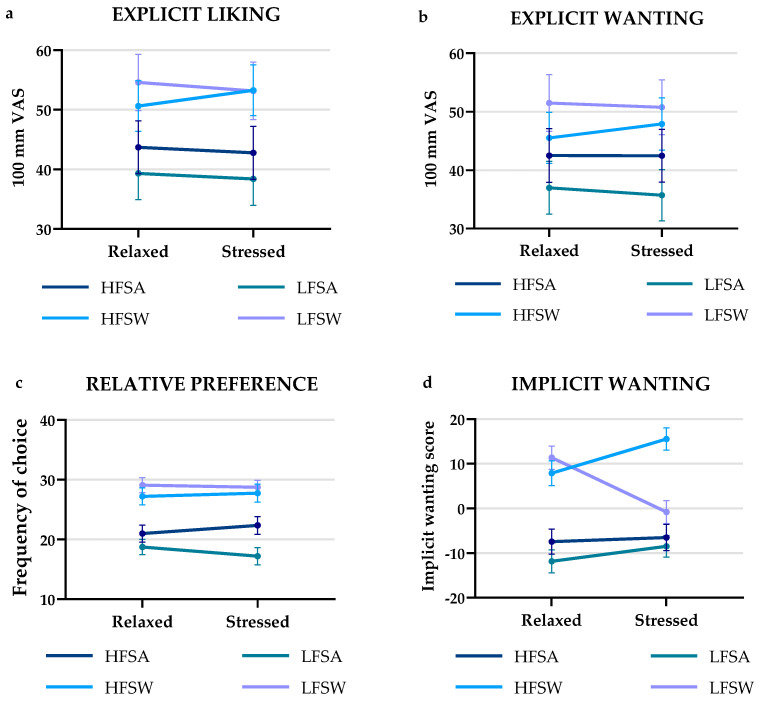
Interaction plots for (**a**) explicit liking (mean ± SEM), (**b**) explicit wanting (mean ± SEM), (**c**) relative preference (mean ± SEM) and (**d**) implicit wanting (mean ± SEM), for the four different food categories. HFSA: high-fat savory; HFSW: high-fat sweet; LFSA: low-fat savory; LFSW: low-fat sweet.

Mean ratings of explicit liking for the four food categories ranged from 38.41–54.57. The ratings can be seen in Table 4 and Figure 4a. The sweet foods were in general rated higher than the savory for both conditions (Relaxed; *p* = 0.016, Stressed; *p* = 0.020), whereas no difference was found in ratings of high-fat foods vs. low-fat foods. In addition, no effect was found of the two conditions on rating of explicit liking for any of the four combined food categories.

For explicit wanting, the four food categories were rated from 35.71–51.49. Mean ratings can be seen in Table 4 and Figure 4b. Again, the sweet foods were rated higher than the savory for both conditions (Relaxed; *p* = 0.027, Stressed; *p* = 0.010), and no difference was found between the high-fat and low-fat foods in rating of explicit wanting. Lastly, no effect could be detected of the stressed vs. relaxed conditions on rating of explicit wanting for any of the four food categories.

#### 3.2.2. Relative Preference

Mean frequency of counts for each combined food category can be seen in Table 4 and Figure 4c. The sweet foods were chosen more than the savory foods for both conditions (Relaxed; *p* = 0.008, Stressed; *p* = 0.011), and there was no difference in choice of high-fat foods over low-fat foods. The two conditions had no effect on relative preference for any of the four combined food categories.

#### 3.2.3. Implicit Wanting

Implicit wanting score ranges can be seen in Table 4, whereas Figure 4d shows mean implicit wanting scores for all four food groups. Sweet foods had a higher implicit wanting score than savory foods (Relaxed; *p* = 0.004, Stressed; *p* = 0.010), and again no difference was detected in regards to high-fat foods over low-fat foods. The stressed condition proved to have a positive effect on implicit wanting scores for the HFSW food category (*p* = 0.006, r = 0.464)) and a negative effect on the LFSW (*p* < 0.001, r = 0.779)) food category (Figure 4d), thus indicating a shift from LFSW foods towards HFSW foods when being stressed.

#### 3.2.4. Actual Snack Choice

The results of the participants’ actual choice of snacks, representing each of the four food categories of the LFPQ, demonstrated effects of the two conditions. Overall, the sweet snacks were chosen more in both conditions (Relaxed; *p* = 0.004, Stressed; *p* = 0.018), and the high-fat snacks were also chosen more in the stressed condition (*p* = 0.045). There were no differences between choice of high-fat vs. low-fat snacks in the relaxed condition. More specifically, the chocolate bar, signifying the HFSW food category, was chosen by 49% of the participants in both conditions. The banana (LFSW) was chosen by 26% of the participants in the relaxed condition, whereas only 14% chose it in the stressed condition (*p* = 0.001). The crackers (LFSA) and potato chips (HFSA) were chosen at equal times (*n* = 4; 11%) for both conditions. Nonetheless, significant differences could still be determined for both snack categories (*p* < 0.001 for both), as the paired McNemar’s test compares the composition of participants who chose the two snack products, and not the frequency of count. Moreover, some participants chose to not take a snack, with more doing so in the stressed condition (*n* = 5; 14%) as compared to the relaxed (*n* = 1; 3%, *p* < 0.001). Overall, 46% of the participants did not change their snack choice between the two conditions, thus revealing that 54% did indeed change direction. In terms of direction of snack choice change, the main change to be detected was that 14% (*n* = 5) chose the option of not choosing a snack. Furthermore, a change was seen from the LFSW towards the HFSW snack choice, with 9% (*n* = 3) of the respondents choosing to do so.

For the two separate test days, no differences could be seen in frequency of choice between the low-fat vs. high-fat snacks for any of the test days; however, when comparing the frequencies regarding sweet vs. savory snacks a difference could be detected on Test Day 2 (*p* < 0.001), with more people choosing the sweet snacks. The distribution of snack choice by the order of the two test days looked like this: on Test Day 1, most participants chose the chocolate bar (HFSW, 43%), followed by the banana (LFSW, 17%), the potato chip (HFSA, 17%), the crackers (LFSA, 11%) and ‘no choice’ option (11%). For Test Day 2, the results were that 54% chose the chocolate bar (HFSW), 23% the banana (LFSW), 11% the cracker (LFSA) and 6% chose the potato chip (HFSA) and 6% no snack. Significant differences were found for the LFSA, HFSA and LFSW snacks, as well as the ‘no choice’ option (*p* < 0.001 for all) between the test days. Again, the tests analyzed composition of participants rather than frequency, meaning that the significant differences are an expression of changes made by the individual participants between the two test days. No effect was found of test day order on the chocolate bar (HFSW) snack. In terms of direction of changes from Test Day 1 to Test Day 2, the biggest change was from the potato chip (HFSA) snack towards the chocolate bar (HFSW) snack with 14% choosing to do so.

## 4. Discussion

In light of the current reported health state of Danes and other western societies with increasing obesity, decreasing mental health and increasing levels of perceived stress [60,61], the relationship between stress and eating behavior observably needs more attention if we are to gain a better understanding of the underlying mechanisms that are at play when making food choices. With this study, the effect of perceived stress on food pleasure and eating behavior has been investigated by use of the LFPQ, and thus distinct measures of food reward as well as actual snack choice behavior. More specifically, the study investigated the effect of acute psychosocial stress on explicit liking, explicit wanting, relative food preference, implicit wanting and actual snack choice. Overall, the study found that there was no effect of acute stress vs. being relaxed on explicit liking, explicit wanting or relative preference for the four food categories. For implicit wanting scores, an effect was detected on the HFSW food group, with increasing scores for the stressed condition; and oppositely, decreasing scores were found for the LFSW food category when being stressed. Actual snack choice showed that in general the sweet snacks were preferred in both conditions, as well as a preference for the high-fat snacks in the stressed condition. The HFSW snack was the most popular choice for both conditions. Furthermore, differences between the two conditions for the LFSW, LFSA and HFSA snack food categories, as well as for the ‘no choice’ option were detected; however, the same results were found when testing for an effect of the order of snack choice.

### 4.1. ‘Stressed’ Is ‘Desserts’ in Reverse for Some

In general, it was found for all LFPQ measures that sweet foods were preferred over savory regardless of condition, whereas no differences could be detected in relation to fat content. This result implies a sweet bias and may be explained by the general preference for sweet foods of Danish consumers, especially in the context of snack meals [62,63].

As in line with previous literature, the results of explicit liking, explicit wanting and relative preference followed the same pattern for all food categories [25,40,46]. Explicit liking as an expression of consummatory pleasure thereby does not seem to change regardless of arousal level, confirming initial hypotheses. Likewise, neither does explicit wanting, which expresses a conscious cognitive desire for a specific food group. These results indicate that being under the influence of acute stress is perhaps not enough to change perceived expected reward of foods, possibly because these subjective sensations can be linked to more stable individual preferences for specific foods [25,50]. The nature and longevity of the stress experience could be an important factor in this, as the strain felt after completing the stress-inducing cognitive task could have been regarded as manageable despite the high arousal levels. Thus, activation of the sympathetic nervous system may not have occurred to a high enough degree, where it affects conscious decision making. At the same time, these results could also be an expression of people’s ability to make consistent autonomous decisions, rather than their true subjective experience in the moment, or that it simply could have been difficult to make a distinction between the two explicit measures [25,33]. Moreover, previous studies using the LFPQ have shown that the explicit pleasure measures often correspond with each other, thus emphasizing the need for implicit measures [25,46].

The results of the implicit wanting scores, nonetheless, were affected by the strain of the cognitive tasks, thereby proving that the unconscious desire for specific foods can be altered. As it is hypothesized that it is these unconscious cravings or desires that truly govern our food choices and eating behaviors, these insights confirm what previous studies have shown; namely that stress, regardless of type or longevity, has the potential to change eating behaviors [18,26,27,35,64]. Furthermore, it was the implicit wanting for HFSW foods that markedly increased during the stressful task, once again confirming the results of previous studies showing that stressed individuals may have an affinity for highly palatable foods [10,14,18,20]. Concurrently, implicit wanting for the LFSW foods decreased, thereby indicating that the perceived calorie density of food is an important factor to consider when seeking to understand the underlying mechanisms of stress-induced eating. Increased consumption of highly ‘palatable’ foods has long been linked to stress and emotional eating [12,19,51]. However, the term ‘palatability’ can refer to foods both high in subjective liking and high sugar and fat contents. An univocal conception of the term therefore does not seem to exist, which leads to confusion of whether it is the macronutrient content of a food, its hedonic properties or a mix of both that defines it as ‘highly palatable’ [18,65]. Regardless of the term definition, acute stress can significantly alter eating behavior, with some studies showing a decreased food intake, and others showing an increased intake, especially of highly palatable, calorie-dense foods [16,65,66].

The actual snack choice made by the participants represented an explicit food-choice situation. It was expected that the results of this would mimic the results of the explicit liking and wanting of the LFPQ, and to some degree they did, as the HFSW snack was chosen most frequently for both conditions. When taking a closer look into the changes made by the participants going from the relaxed to the stressed condition, most participants did not make a change (46%), whereas 31% stayed with the HFSW snack. The biggest change to be seen in snack choice was from choosing the LFSW snack in the relaxed condition to choosing the HFSW snack in the stressed condition, with 9% of the participants making this shift. This result reflects the results of the implicit wanting scores. Thereby, it seems possible that the implicit wanting as a measure of unconscious pleasure demonstrates its ability to change people’s food choice, as well as underpins that acute psychosocial stress can alter food choice in a direction of more calorie-dense foods. Thus, these results confirm observations made in previous studies of stress causing a higher intake of palatable, calorie-dense foods [10,24,52]. Studies determining the effect of the cognitive tasks via endocrine measures, such as salivary cortisol levels, are needed to clarify if these results were an actual consequence of an activation of the HPA axis. Nonetheless, one should also bear in mind when reflecting upon these results that the number of participants was rather low (*n* = 35 in total), and thus 9% represents a total of three participants. Furthermore, as an effect of test day was likewise observed, further analyses with more subjects are needed to clarify the effect size of condition as compared to test day.

Taken as a whole, the results indicate that when under the influence of acute psychosocial stress cravings for highly palatable foods increase for some people, even though this is not what they explicitly express, while for others an experience of loss of appetite may be expressed. Further research could usefully explore this differentiation, as one might hypothesize that each individual perceives pleasure from food in their own way, and thereby exhibit different food-related reactions to stress on an individual level.

### 4.2. Implications

The current study confirms links between stress, the reward pathways and eating behavior previously alluded to in the literature [16,18,20]; however, these links have not previously been investigated by detailed measures of implicit and explicit liking and wanting. Thereby, the link between acute psychosocial stress and these distinct reward mechanisms have been highlighted for the first time, providing insights for further research in the field of food reward, stress and consumer behaviors. The measure of implicit wanting has through this study underlined its justification, as an important concept in food reward research. Further investigation of this concept in other consumer groups, such as people with anhedonic traits or inability to feel pleasure, would be highly interesting, to further understand how this specific aspect of food cognition can affect food choice and eating behavior.

As stress is becoming an ever-increasing health problem that appears to be grounded in the very structure of modern life, preventing stress conditions seems an unattainable task [1,2]. Nonetheless, prevention of some of the repercussions of this condition should be of high importance both in research as well as in public health regulations. Further research into psychobehavioral effects of both acute and chronic stress on food choice and eating behavior is therefore required, if possible solutions for consequences of stress are to be found. Moreover, this study can offer valuable insights for the public health sector, and potentially inform efforts to prevent stress-induced weight gain and obesity. On a practical level, practitioners could use these results to guide patients suffering from periods of stress about the possible weight fluctuations this condition can cause. Oppositely, on a societal level, the stigma surrounding people who struggle with stress-induced eating could be remedied in terms of having a better understanding of the internal processes that can happen, when a person is exposed to stressors. Alleviation of stigmas in relation to overeating and obesity could in itself reduce some of the psychosocial stress many people experience [14,39,67,68].

### 4.3. Strengths and Limitations of the Study

To the author’s knowledge, no other studies have investigated the relation between stress, food reward and explicit liking, explicit wanting and implicit wanting in a human study by combining both physiological, subjective, and behavioral measurements, as well as both explicit and implicit measures. This study therefore offers unique insights into how acute stress in practice may affect reward mechanisms in a normal-weight sample with both genders equally represented and begins the process of showing how people could respond in terms of food choice and eating behavior. The study highlights how even short-term stressful conditions can alter eating behavior and appetite. Implicit measures can enlighten areas that questionnaires or other explicit measures cannot, thus giving reliable insights into the psychophysiological aspects of eating behavior; insights that in the light of the general health of many westernized populations may have the potential to be beneficial.

This study had certain limitations such as the technical lab setup, which may well have influenced the participants. Being part of a research study in a controlled environment, including having electrodes attached to one’s hand while performing a cognitive task, may have added stress to the experience, regardless of which cognitive task they had on the day. The nonsignificant results of the GSR measurements may very likely be a result of this. Nonetheless, the subjective ratings on VAS of stress and emotional levels proved the participants did indeed experience an effect of the stress condition. In addition, the length of each test day (approx. 45 min per test day) may have caused some degree of exhaustion. On the other hand, it was expected that the composition and randomization of the tasks and food images would alleviate this. The COVID-19 situation of the time of the data collection also made it difficult to recruit and plan the test days for the participants, especially in terms of matching the time of day for the two test days. Time of day for testing varied for the participants, meaning that some had the tests in the morning, while others conducted the test after lunch. Naturally, this could have influenced the participants’ explicit rating of the food images as well as snack choice. Controlling for these variations was therefore pursued by instructing the participants to follow the same guidelines before arrival for each test day. The instructions were to have a good night’s sleep, not smoke 3 h prior, not engage in any moderate-to-vigorous exercise 2 h prior, and to consume a meal that leaved them comfortably satiated. The researcher verbally confirmed with the participants that they adhered to these instructions before commencing with the trials. Furthermore, participants acted as their own control in the data analysis, thereby further reducing confounding in the data analysis.

## 5. Conclusions

The present study set out to investigate the link between acute psychosocial stressors, perceived pleasure from food, and consequently, food behavior in humans, by help of both physiological, subjective and behavioral measurements. As an exploratory element, the effect on actual snack choice was likewise included. By doing so, the ‘stress-eating behavior’ relationship was investigated, not only by consumption levels or food choice of specific food groups, but by the underlying mechanisms of anticipatory and experienced food pleasure. The most obvious finding to emerge from this study was that explicit ratings of food reward, both in terms of liking, wanting and relative preference, were not affected by acute psychosocial stressors; however, when it came to the implicit ratings of wanting an effect could be detected, with decreasing scores for the LFSW foods, and increasing scores for the HFSW foods. More studies are needed to conclude on the effect of psychosocial stress on actual snack choice, as results confirmed that acute psychosocial stress changed selection of snack choice from low-fat sweet to high-fat sweet or no snack choice, but an effect of test-day order was likewise found. Taken together, these results suggest that when under the influence of acute psychosocial stress, cravings for highly palatable foods increase for some people, even though this is not what they explicitly express, while for others, an experience of loss of appetite may be exhibited. Overall, this study strengthens the evidence that acute stress can affect eating behavior, and it can by at least two different stress-response pathways: one that increases affinity for high-fat sweet foods, and one where appetite altogether may be inhibited.

The study contributes to our understanding of how acute stressors can affect eating behavior, by looking deeper into the individual reward mechanisms and how these are affected. Insights from this study could be relevant to further comprehend why many people in postmodern society experience loss of control of their diet. Further work is needed to fully understand the implications of the reward pathways in relation to human eating behavior and explicitly stress-induced eating.

## Figures and Tables

**Table 1:** Participant characteristics.

Characteristics	
n_total_	35
Males/females (%)	17/18 (49%/51%)
Age (years) *	21.71 ± 2.04 (18–25)
Educational level	
- Primary school (%)	4 (11%)
- High school (%)	25 (71%)
- Short Higher Education (%)	1 (3%)
- Medium Higher Education (%)	4 (11%)
- Long Higher Education	1 (3%)
Socioeconomic status	
- Student (%)	28 (80%)
- Unemployed (%)	1 (3%)
- Employee (%)	6 (17%)
BMI (kg/m^2^) ^1^,*	22.14 ± 1.67 (19–25)
PSS-10 ^2^,*	16.11 ± 5.18 (7–27)
- Low stress; 0–13 (%)	12 (34%)
- Moderate stress; 14–26 (%)	22 (63%)
- High stress; 27–40 (%)	1 (3%)

* Mean ± standard deviation (range), ^1^ BMI: body mass index, ^2^ PSS-10: perceived stress scale [42,43].

**Table 2:** Macronutrient content of the foods chosen for the LFPQ image array.

	E%				E%		
	Pro	Carb	Fat		Pro	Carb	Fat
**HFSA**				**LFSA**			
Potato chips	5	36	55	Salad	18	50	19
Cheese on cracker	22	34	44	Cucumber	27	65	8
Mixed nuts	16	14	70	Pretzels	11	75	8
Quiche	15	24	59	Turkey on crispbread	31	63	6
**HFSW**				**LFSW**			
Donut	5	41	50	Mixed berry salad	8	77	4
Milk chocolate	6	37	53	Skittles	0	84	9
Blueberry muffin	5	44	58	Wine gums	8	86	1
Cinnamon roll	5	38	54	Banana	5	84	4

E%: energy percentage; HFSA: high-fat savory; HFSW: high-fat sweet; LFSA: low-fat savory; LFSW: low-fat sweet.

**Table 3:** Macronutrient content of snack-food selection.

		E%		
	kJ/100 g	Pro	Carb	Fat
**HFSA:** Potato chips	2149	5	44	50
**HFSW:** Chocolate bar	2018	7	42	51
**LFSA:** Wholegrain crackers	1856	13	54	30
**LFSW:** Banana	396	5	90	2

E%: energy percentage; HFSA: high-fat savory; HFSW: high-fat sweet; LFSA: low-fat savory; LFSW: low-fat sweet.

**Table 4:** Mean (±SD) Leeds Food Preference Questionnaire outputs for the different food categories, relaxed and stressed condition.

	**Explicit Liking (mm)**			**Explicit Wanting (mm)**		
	Relaxed	Stressed	*p*-value	Relaxed	Stressed	*p*-value
HFSA	43.70 (±26.18)	42.76 (±26.25)	NS	42.50 (±27.32)	42.46 (±26.72)	NS
LFSA	39.31 (±26.04)	38.41 (±26.26)	NS	36.97 (±26.65)	35.71 (±25.90)	NS
HFSW	50.63 (±25.11)	53.26 (±25.26)	NS	45.53 (±25.86)	47.89 (±26.49)	NS
LFSW	54.57 (±27.96)	53.14 (±28.54)	NS	51.49 (±28.77)	50.76 (±27.76)	NS
	**Implicit wanting (range)**			**Frequency of choice (count)**		
	Relaxed	Stressed	*p*-value	Relaxed	Stressed	*p*-value
HFSA	−38–36	−25–41	NS	20.97 (±8.40)	22.34 (±8.83)	NS
LFSA	−31–24	−31–116	NS	18.74 (±7.61)	17.20 (±8.49)	NS
HFSW	−28–34	−14–57	0.006	27.20 (±8.52)	27.74 (±8.88)	NS
LFSW	−18–46	−13–71	<0.001	29.09 (±7.42)	28.71 (±6.93)	NS

HFSA: high-fat savory; HFSW: high-fat sweet; LFSA: low-fat savory; LFSW: low-fat sweet; NS: nonsiginificant.

## Data Availability

The datasets generated for this study are available on request to the corresponding author.

## Supplementary Materials

The following supporting information can be downloaded at: https://www.mdpi.com/article/10.3390/foods11121756/s1, Table S1: Images used in the Leeds Food Preference Questionnaire; Table S2: Mean GSR amplitude levels and peaks/min at baseline, during performance of the cognitive task and during performance of the Leeds Food Preference Questionnaire.
