# Supplementary material for: Why Being ‘Stressed’ Is ‘Desserts’ in Reverse—The Effect of Acute Psychosocial Stress on Food Pleasure and Food Choice
Source: Foods. 2022 Jun 15;11(12):1756. doi: 10.3390/foods11121756 (PMC9222595; doi:10.3390/foods11121756)
Supplement: Supplementary file 1 [file foods-11-01756-s001.zip › foods-1729309-SI.pdf]

Supplementary materials

Table S1. Images used in the Leeds Food Preference Questionnaire. There were four images grouped together to represent each of four food categories. HFSW: High-fat sweet, LFSW: Low-fat sweet, HFSA: High-fat savory, LFSA: Low-fat savory

Food category				
HFSW	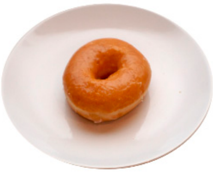	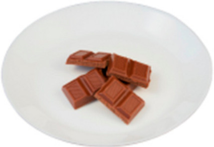	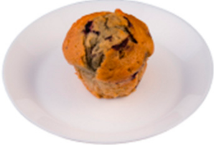	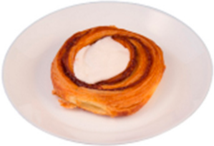
LFSW	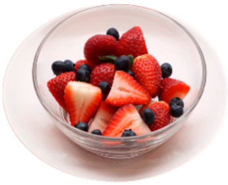	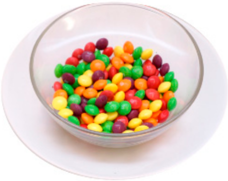	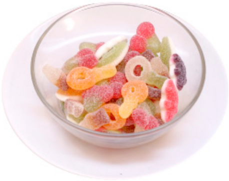	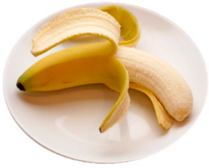
HFSA	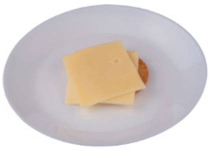	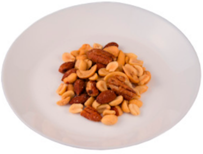	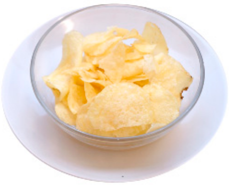	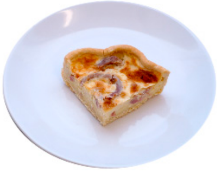
LFSA	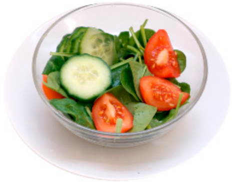	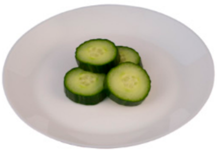	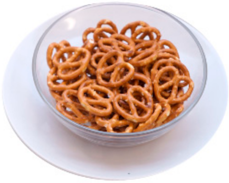	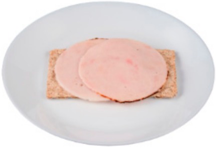

Table S2. Mean (±SD) GSR amplitude levels and peaks/min at baseline, during performance of the cognitive task and during performance of the Leeds Food Preference Questionnaire.

	**Condition**		**Result of linear mixed effects ANOVA**	
**GSR measures**	**Relaxed**	**Stressed**	**F value**	**P value**
**Baseline, mean (±SD) amplitude**	9.69 (±3.91)	9.63 (±3.83)	0.021	0.886
**Cognitive task, mean (±SD) amplitude**	13.13 (±4.29)	13.63 (±4.00)	0.169	0.686
**Cognitive task, peaks/min (±SD)**	4.51 (±1.96)	3.65 (±1.54)	4.112	0.053
**LFPQ, mean (±SD) amplitude**	13.36 (±4.40)	13.53 (±3.98)	0.039	0.844
**LFPQ, peaks/min (±SD)**	2.58 (±1.38)	2.09 (±1.04)	1.714	0.204

LFPQ: Leeds Food Preference Questionnaire
